# Efficacy of chemotherapy combined with surgical resection for gastric cancer with synchronous ovarian metastasis: A propensity score matching analysis

**DOI:** 10.1002/cam4.6362

**Published:** 2023-07-30

**Authors:** Jingquan Fang, Xingmao Huang, Xiangliu Chen, Qi Xu, Tengjiao Chai, Ling Huang, Han Chen, Hang Chen, Zeyao Ye, Yian Du, Pengfei Yu

**Affiliations:** ^1^ Department of Gastric Surgery, Zhejiang Cancer Hospital, Institute of Basic Medicine and Cancer (IBMC) Chinese Academy of Sciences Hangzhou China; ^2^ Zhejiang Chinese Medical University Hangzhou China; ^3^ Wenzhou Medical University Wenzhou China; ^4^ Department of Medical oncology, Zhejiang Cancer Hospital, Institute of Basic Medicine and Cancer (IBMC) Chinese Academy of Sciences Hangzhou China

**Keywords:** chemotherapy, gastric cancer, ovarian metastasis, prognosis, surgical treatment

## Abstract

**Background:**

Ovarian metastasis from gastric cancer (GC) is characterized by aggressive biological behavior and poor outcome. Currently, there is no standard treatment mode for such patients. Thus, we evaluated the efficacy of conversion therapy in patients with synchronous ovarian metastasis from GC in this study.

**Methods:**

About 219 GC patients with ovarian metastasis in 2011–2020 were enrolled. Two groups were established based on the different treatment: the conversion therapy group (chemotherapy combined with surgical resection, CS group) and the non‐conversion therapy group (NCS group). Propensity score matching (PSM) was used to analyze the efficacy of different treatment modes on the prognosis of these patients.

**Results:**

Ninety‐two patients were included according to PSM results, with 46 patients each in CS and NCS groups. The median overall survival (OS) in the CS group was notably better than that in the NCS group (*p* < 0.001). Twenty‐six patients (56.52%) in the CS group achieved R0 resection, and they had a better prognosis (*p* = 0.003). Compared with patients who underwent simultaneous gastrectomy and ovarian metastasectomy (CSb group), those who underwent ovarian metastasectomy before systemic chemotherapy (CSa group) had a higher R0 resection rate (*p* = 0.016) and longer survival time (*p* = 0.002). A total of 38 patients (41.30%) across both groups received hyperthermic intraperitoneal chemotherapy (HIPEC), and these patients had a better survival (*p* = 0.043).

**Conclusion:**

The conversion therapy is safe and effective for patients with synchronous ovarian metastasis from GC and can improve their prognosis. However, our results need to be confirmed by more randomized controlled clinical studies.

## 1 INTRODUCTION

Gastric cancer (GC) is among the most common gastrointestinal malignancies as well as the second leading cause of cancer‐related deaths worldwide.^1^ GC patients always have a poor prognosis because of metastasis and high recurrence rate. Krukenburg tumor refers to the ovary metastatic tumor of the ovary which primarily arises from gastrointestinal tract and GC is the most common primary source,^2^, ^3^, ^4^ which tends to be associated with poor treatment outcomes.^5^, ^6^ Currently, systemic chemotherapy is still the main choice in the treatment for GC patients with ovarian metastases, but its efficacy is still unsatisfactory with only 7–14 months of survival.^6^, ^7^

Conversion therapy has come to be increasingly adapted in the treatment of advanced GC in recent years. After a comprehensive treatment like chemotherapy, the primary lesions and metastases of GC can be well controlled, and this may present an opportunity for the use of radical surgery, which would prolong the survival of these patients.^8^, ^9^, ^10^ Several studies have investigated the benefit of ovarian metastasectomy for GC patients with synchronous ovarian metastases.^11^, ^12^, ^13^ However, the application of conversion therapy in ovarian metastasis from GC and the value of the primary GC resection after ovarian metastasectomy are still unclear. There is no consensus on the ideal treatment strategy for these patients.

In this study, propensity score matching (PSM) was used to balance the baseline characters and analyzed the efficacy of different treatment modalities and their influence on the prognosis of patients in pursuit of identifying a scientific and effective treatment model for patients with synchronous ovarian metastasis from GC.

## 2 MATERIALS AND METHODS

### 2.1 Patients

**FIGURE 1:**
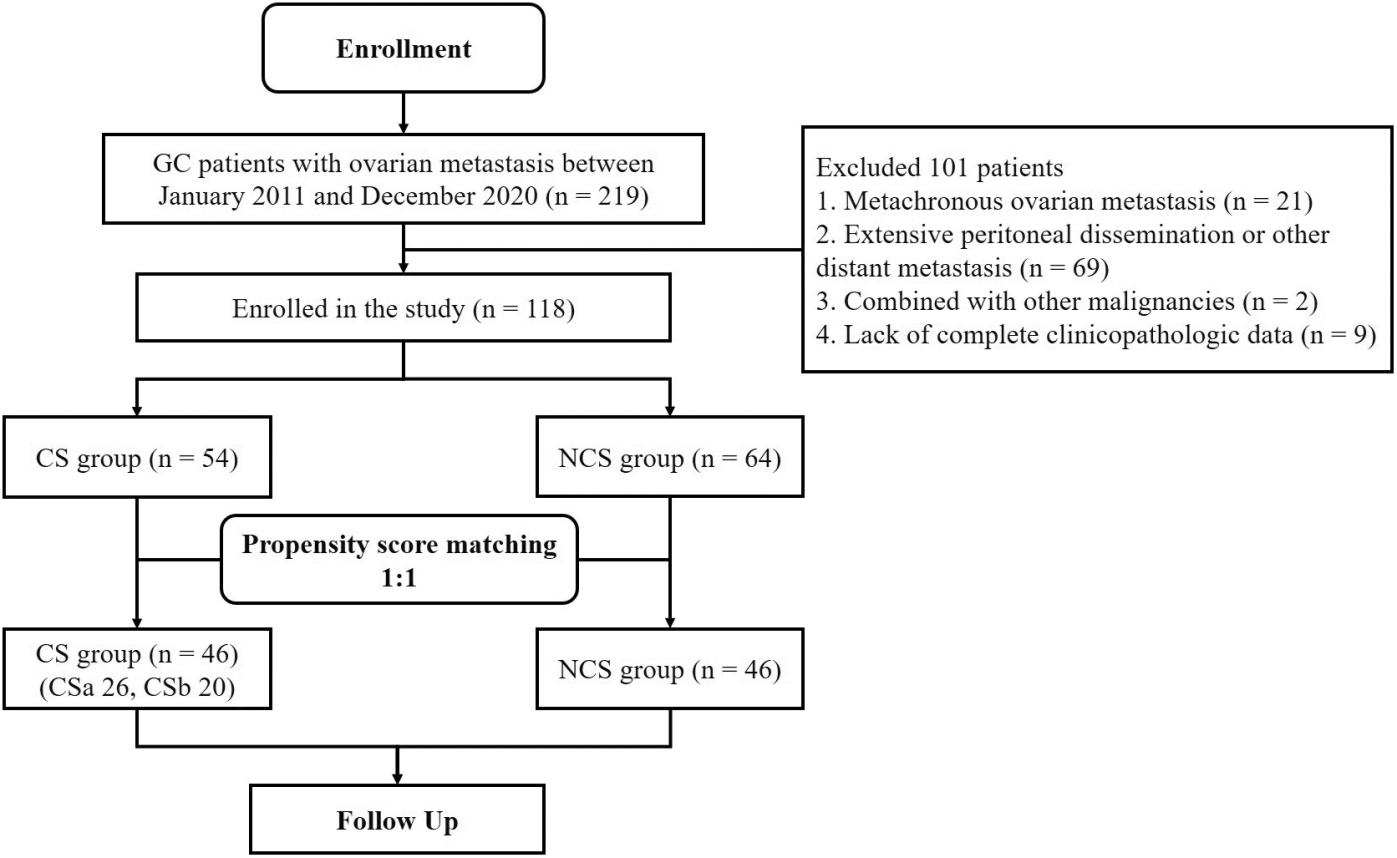
Flow diagram of the patient selection process. CS, conversion surgery; CSa, ovarian metastases were resected before systemic chemotherapy; CSb, ovarian metastases were resected after systemic chemotherapy; GC, gastric cancer; NCS, non‐conversion surgery.

The clinicopathologic data were retrospectively reviewed in 219 GC patients with ovarian metastasis who underwent gastrectomy and metastasectomy or systemic chemotherapy as the initial treatment in Zhejiang Cancer Hospital (Hangzhou, China) from January 2011 to December 2020. The study design is presented in Figure 1.

Patients were enrolled according to the following criteria: (1) histologically confirmed primary gastric/esophagogastric junction adenocarcinoma; (2) age 18–75 years; (3) synchronous ovarian metastasis from GC (ovarian metastases were diagnosed simultaneously with GC or confirmed within 6 months after GC diagnosis) without peritoneal metastasis or with limited peritoneal metastasis (P0/1, according to the Japanese Gastric Cancer Treatment Guideline, fifth edition^14^); (4) Eastern Cooperative Oncology Group performance status (PS) 0–1; and (5) adequate organ function.

The exclusion criteria included: (1) with extensive peritoneal dissemination or other distant metastasis; (2) metachronous ovarian metastasis from GC (ovarian metastases were diagnosed more than 6 months after GC diagnosis or surgery); (3) combined with other malignancies; and (4) lack of complete clinicopathologic data.

According to the above criteria, 101 cases were excluded, and 118 cases were finally included in the analysis (Table 1). Clinicopathologic factors like age, metastasis size, tumor marker level, pathological type, differentiation, T and N stage (AJCC eighth edition), treatment modality, and survival time were collected and collated for analysis. This study has been approved by the Ethics Committee of Zhejiang Cancer Hospital (no. IRB‐2022‐279).

**TABLE 1:** Baseline characteristics of 118 GC patients with synchronous ovarian metastasis before PSM.

Variable	CS Group (*n* = 54)	%	NCS Group (*n* = 64)	%	*χ* ^2^‐value	*p*‐value
Age (years)						
<50	35	64.8	41	64.1	0.007	1.000
≥50	19	35.2	23	35.9		
Tumor size (cm)						
<5	15	27.8	20	31.3	0.169	0.692
≥5	39	72.2	44	68.8		
Laterality						
Unilateral	12	22.2	18	28.1	0.538	0.528
Bilateral	42	77.8	46	71.9		
cT‐stage						
≤T2	4	7.4	10	15.6	1.891	0.254
>T2	50	92.6	54	84.4		
cN‐stage						
N0/1	36	66.7	41	64.1	0.088	0.847
N2/3	18	33.3	23	35.9		
Tumor location						
Upper	5	9.2	9	14.1	0.650	0.723
Middle	30	55.6	34	53.1		
Lower	19	35.2	21	32.8		
Ascites						
Negative	39	72.2	31	48.4	7.792	**0.014**
Positive	15	27.8	33	51.6		
Differentiation						
Poorly/undifferentiated	50	92.6	62	96.9	1.113	0.410
Well/moderately	4	7.4	2	3.1		
Signet‐ring cells						
Negative	10	18.5	20	31.3	2.504	0.139
Positive	44	81.5	44	68.8		
Peritoneum metastasis						
Negative	24	44.4	14	21.9	6.833	**0.011**
Positive	30	55.6	50	78.1		
Serum CEA (U/mL)						
Normal	40	74.1	46	71.9	0.072	0.838
>5	14	25.9	18	28.1		
Serum CA199 (U/mL)						
Normal	39	72.2	28	43.8	9.675	**0.003**
>37	15	27.8	36	56.3		
Serum CA125 (U/mL)						
Normal	25	46.3	13	20.3	9.057	**0.003**
>35	29	53.7	51	79.7		

Abbreviations: CA125, carbohydrate antigen 125; CA199, carbohydrate antigen199; CEA, carcinoembryonic antigen.

Values in bold indicate statistical differences.

### 2.2 Treatment and evaluation

According to the treatment modality, two groups were established: conversion therapy group (CS group) and non‐conversion therapy group (NCS group). Before the initial treatment, imaging examination was performed in all patients to assess the severity of disease and resectability. Surgical resection was performed according to consensual opinion after comprehensive assessment of patient by a multidisciplinary team.

The NCS group received palliative chemotherapy with or without exploratory surgery (some patients were considered resectable according to imaging evaluation, but the radical resection could not be obtained at the time of surgery, and these patients underwent exploratory surgery). The CS group received chemotherapy plus surgery (gastrectomy and metastasectomy). In addition, we divided the CS group into CSa group (ovarian metastases were resected before systemic chemotherapy) and CSb group (ovarian metastases were resected after systemic chemotherapy).

#### 2.2.1 Systemic chemotherapy

PS regimen: intravenous paclitaxel (150 mg/m^2^; day 1) plus S‐1 dose was calculated on the basis of body surface area (40 mg/day, if <1.25 m^2^; 50 mg/day, if 1.25–1.50 m^2^; 60 mg/day, if ≥1.50 m^2^; days 1–14) for a 21‐day cycle.

#### 2.2.2 Surgical treatment

All patients underwent comprehensive evaluation 4–6 weeks after their last chemotherapy session. Surgical resection was performed when the tumor was well controlled, and it included gastrectomy, D2 lymph node dissection and metastasectomy.

#### 2.2.3 Hyperthermic intraperitoneal chemotherapy

Some patients received hyperthermic intraperitoneal chemotherapy (HIPEC) after the initial exploratory surgery: paclitaxel (75 mg/m^2^) with 3 L of 0.9% saline was heated to 43.0°C ± 0.3°C and infused into the peritoneal cavity via an automatic HIPEC device (BR‐TRG‐II, Bright Medical Technology Co., Ltd.) for a circulation about 60 min.

#### 2.2.4 Evaluation

The Clavien–Dindo severity classification^15^ was performed to categorize postoperative complications, and the Common Terminology Criteria for Adverse Events, Version 5.0 (CTCAE v5.0)^16^ was performed to evaluate adverse events. Histological tumor regression grade (TRG)^17^ was used to assess tumor regression in surgical specimens after chemotherapy.

### 2.3 Follow‐up

Follow‐up was performed by regular outpatient reexaminations and telephonic follow‐ups (once/3 months in year 1–2; once/6 months, in year 3–5; once per year, thereafter). Overall survival (OS) was defined as the time of pathological diagnosis of GC and the cutoff date was June 30, 2022.

### 2.4 PSM analysis

PSM was calculated based on a logistic regression model to balance the baseline characters between the groups. According to the analysis results and prognostic factors reported already,^18^, ^19^ we selected the maximum diameter of Krukenberg tumors, the depth of invasion of the gastric tumor, degree of lymph node metastasis, ascites, combined peritoneal carcinomatosis and preoperative serum levels of CA199 and CA125 as matching factors to construct the PSM model. Nearest neighbor matching was performed without replacement at a ratio of 1:1, and a caliper width with a 0.05 standard deviation was specified.

### 2.5 Statistical analysis

All data were analyzed by SPSS 26.0 (IBM Corporation) and statistical significance was defined as a *p* < 0.05. Student's *t*‐test was used for continuous variables and chi‐squared test was used to assess discrete variables. The Kaplan–Meier method and log‐rank test were used for survival analysis. The Cox regression model was used to estimated hazard ratio (HR) and 95% confidence interval (95% CI) in univariable and multivariable analysis.

## 3 RESULTS

### 3.1 Patient characteristics

Ninety‐two patients were enrolled according to the PSM result, including 46 patients in the NCS group while 46 patients in the CS group (CSa group 26 cases, and CSb group 20 cases). The median age at participation was 44.9 years (range, 19–65 years); the primary tumor was poorly differentiated adenocarcinoma in 87 cases (94.57%) and well‐ or moderately differentiated adenocarcinoma in five cases (5.43%). Primary tumor invasion was T1–T2 in 11 cases (11.96%) and T3–T4 in 81 cases (88.04%). Ovarian metastases were bilateral in 69 cases (75.00%) and unilateral in 23 cases (25%). The maximum diameter of Krukenberg tumors was <5 cm in 20 cases (21.74%) and ≥5 cm in 72 cases (78.26%). Peritoneal metastasis was noted in 61 cases (66.30%) and was absent in 31 cases (33.70%). Baseline characteristics in two groups were presented in Table 2. In those patients with peritoneal metastasis, the median peritoneal cancer index (PCI)^20^ was 10.0 ± 5.1, and there was no statistical difference of PCI between the CS group and NCS group (CS group 8.5 ± 4.9, NCS group 11.1 ± 5.0; *p* = 0.889).

**TABLE 2:** Baseline characteristics of 92 GC patients with synchronous ovarian metastasis after PSM.

Variable	CS Group (*n* = 46)	%	NCS Group (*n* = 46)	%	*χ* ^2^‐value	*p‐*value
Age (years)						
<50	29	63.0	30	65.2	0.047	1.000
≥50	17	37.0	16	34.8		
Tumor size (cm)						
<5	10	21.7	10	21.7	0.000	1.000
≥5	36	78.3	36	78.3		
Laterality						
Unilateral	8	17.4	15	32.6	2.841	0.148
Bilateral	38	82.6	31	67.4		
cT‐stage						
≤T2	3	6.5	8	17.4	2.581	0.197
>T2	43	93.5	38	82.6		
cN‐stage						
N0/1	32	69.6	29	63.0	0.438	0.659
N2/3	14	30.4	17	37.0		
Tumor location						
Upper	3	6.5	7	15.2	1.920	0.383
Middle	27	58.7	23	50.0		
Lower	16	34.8	16	34.8		
Ascites						
Negative	31	67.4	31	67.4	0.000	1.000
Positive	15	32.6	15	32.6		
Differentiation						
Poorly/undifferentiated	43	93.5	44	95.7	0.211	1.000
Well/moderately	3	6.5	2	4.3		
Signet‐ring cells						
Negative	9	19.6	16	34.8	2.691	0.159
Positive	37	80.4	30	65.2		
Peritoneum metastasis						
Negative	20	43.5	11	23.9	3.941	0.077
Positive	26	56.5	35	76.1		
Serum CEA (U/mL)						
Normal	35	76.1	32	69.6	0.494	0.640
>5	11	23.9	14	30.4		
Serum CA199 (U/mL)						
Normal	32	69.6	22	47.8	4.483	0.056
>37	14	30.4	24	52.2		
Serum CA125 (U/mL)						
Normal	19	41.3	10	21.7	4.079	0.072
>35	27	58.7	36	78.3		

Abbreviations: CA125, carbohydrate antigen 125; CA199, carbohydrate antigen199; CEA, carcinoembryonic antigen.

### 3.2 Treatment outcome and prognostic factors

Patients in the NCS group received an average of 3.9 cycles of chemotherapy (range, 2–8 cycles), with 16 patients undergoing exploratory surgery and 14 patients (30.43%) receiving an average of 1.7 cycles of HIPEC (range, 1–3 cycles).

Patients in the CS group received an average of 2.9 cycles of chemotherapy (range, 2–4 cycles) before gastrectomy and 3.8 cycles (range, 2–10 cycles) after the operation. Twenty‐four patients (52.17%) received an average of 2.0 cycles of HIPEC (range, 1–3 cycles). Among the enrolled patients, 26 patients underwent total gastrectomy while 20 patients underwent distal gastrectomy. Five patients (10.9%) received combined visceral resections, which included pancreatectomies in three cases, splenectomy in one case, and colectomy in one case. Notably, 26 patients (56.52%) achieved R0 resection, and our analysis showed that ovarian metastasectomy before systemic chemotherapy (*χ* ^2^ = 6.669, *p* = 0.016) and preoperative N0/1 (*χ* ^2^ = 8.650, *p* = 0.006) were associated with better R0 resection rate. Additionally, 24 patients had tumor degeneration ≤TRG 2 (CSa group 17 cases, CSb group seven cases; *p* = 0.028). The clinicopathologic factors and treatment outcomes of the CS group are presented in Table 3.

**TABLE 3:** Clinicopathologic factors and treatment outcomes of the CS group.

Variable	CSa Group (*n* = 26)	%	CSb Group (*n* = 20)	%	*χ* ^2^‐value	*p*‐value
Age (years)						
<50	17	65.4	12	60.0	0.141	0.765
≥50	9	34.6	8	40.0		
Tumor size (cm)						
<5	3	11.5	7	35.0	3.657	0.077
≥5	23	88.5	13	65.0		
cT‐stage						
≤T2	2	7.7	1	5.0	0.134	1.000
>T2	24	92.3	19	95.0		
cN‐stage						
N0/1	17	65.4	15	75.0	0.494	0.535
N2/3	9	34.6	5	25.0		
Tumor location						
Upper	1	3.8	2	10.0	1.389	0.499
Middle	17	65.4	10	50.0		
Lower	8	30.8	8	40.0		
Ascites						
Negative	16	61.5	15	75.0	0.932	0.365
Positive	10	38.5	5	25.0		
Differentiation						
Poorly/undifferentiated	24	92.3	19	95.0	0.134	1.000
Well/moderately	2	7.7	1	5.0		
Peritoneum metastasis						
Negative	10	38.5	10	50.0	0.612	0.552
Positive	16	61.5	10	50.0		
Cycles of chemotherapy before gastrectomy						
2	6	23.0	7	35.0	4.756	0.093
3	10	38.5	11	55.0		
4	10	38.5	2	10.0		
HIPEC						
Yes	15	57.7	9	45.0	0.730	0.552
None	11	42.3	11	55.0		
TRG grade						
≤2	17	65.4	7	35.0	5.839	**0.028**
>2	6	23.1	12	60.0		
Missing	3	11.5	1	5.0		
Surgical radicalness						
R0	19	73.1	7	35.0	6.669	**0.016**
R1/R2	7	26.9	13	65.0		
Cycles of chemotherapy after gastrectomy						
2–4	20	76.9	13	65.0	0.793	0.511
>4	6	23.1	7	35.0		
Grade 3/4 adverse effects						
Yes	5	19.2	5	25.0	0.221	0.726
None	21	80.8	15	75.0		

Values in bold indicate statistical differences.

### 3.3 Survival outcomes

The median follow‐up duration was 17.6 (range 3–72) months. The median overall survival (mOS) of the 92 patients was 14.0 (95% CI 11.5–16.5) months, and the 1‐year survival rates was 57.0% while the 2‐year survival rates was 24.3%.

**FIGURE 2:**
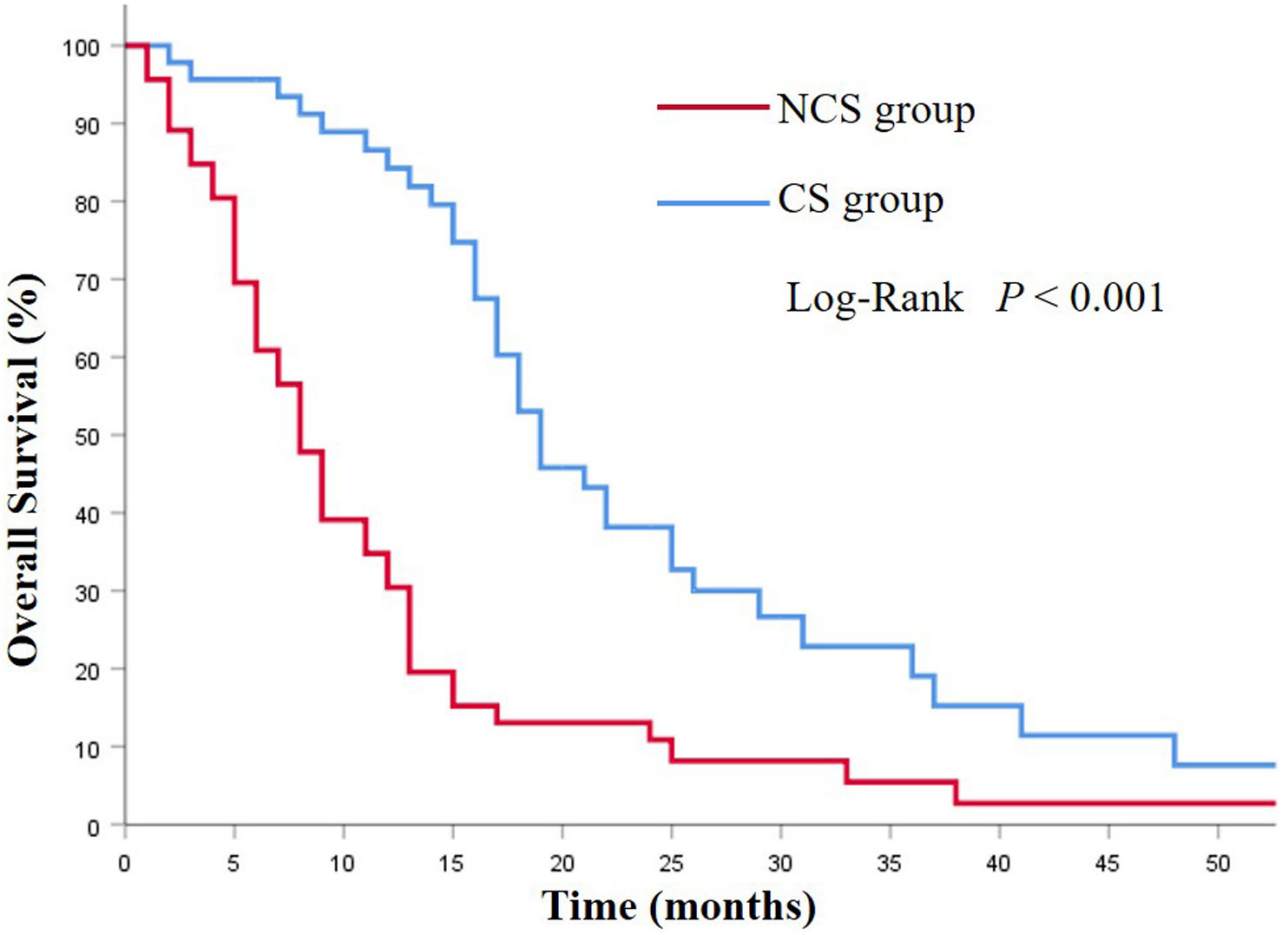
Kaplan–Meier analysis of overall survival in patients with or without conversion therapy.

**FIGURE 3:**
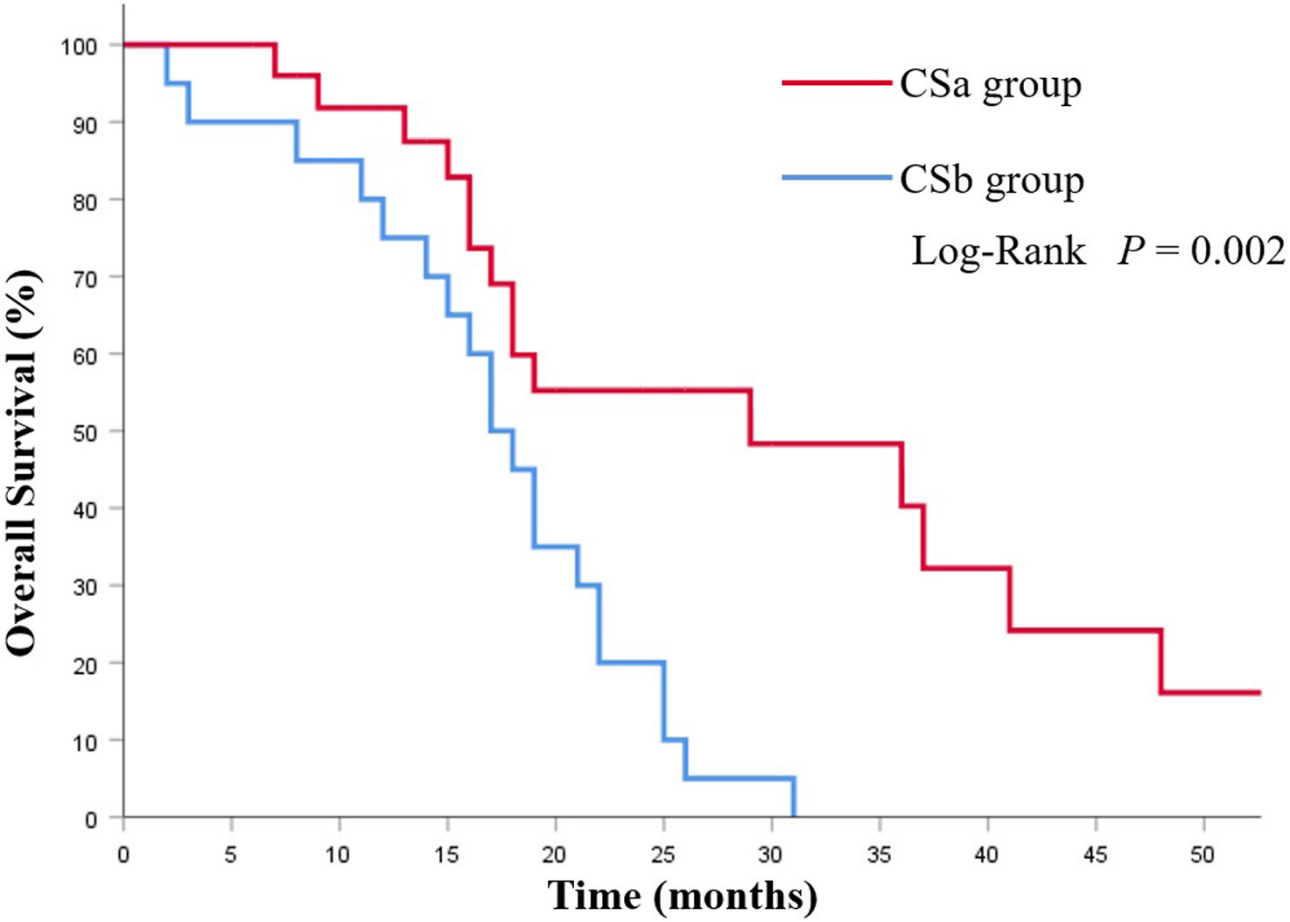
Kaplan–Meier analysis of overall survival in patients with or without metastasectomy of ovarian metastases before systemic chemotherapy.

**FIGURE 4:**
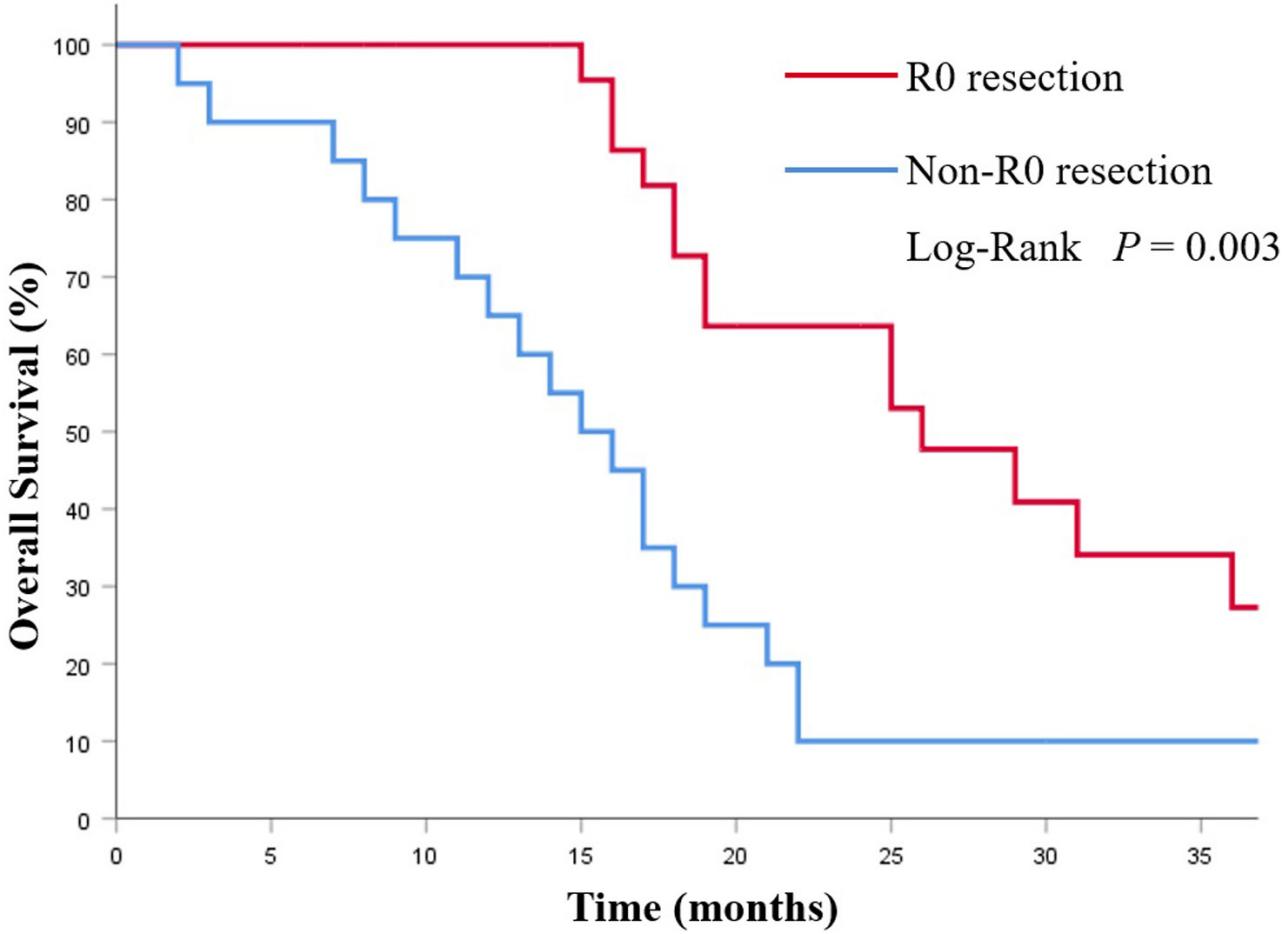
Kaplan–Meier analysis of overall survival in patients with or without R0 resection.

The mOS in the CS group was 19.0 (95%CI 15.4–22.5) months, which was notably longer than that of the NCS group (8.0 months, 95% CI 6.34–9.66 months; *p* < 0.001; Figure 2). Among the CS group, the CSa group had better prognoses compared with the CSb group (29.0 vs. 17.0 months; *p* = 0.002; Figure 3). Additionally, the survival was significantly prolonged among the patients with R0 resection (26.0 months vs. 15.0 months; *p* = 0.003; Figure 4).

**FIGURE 5:**
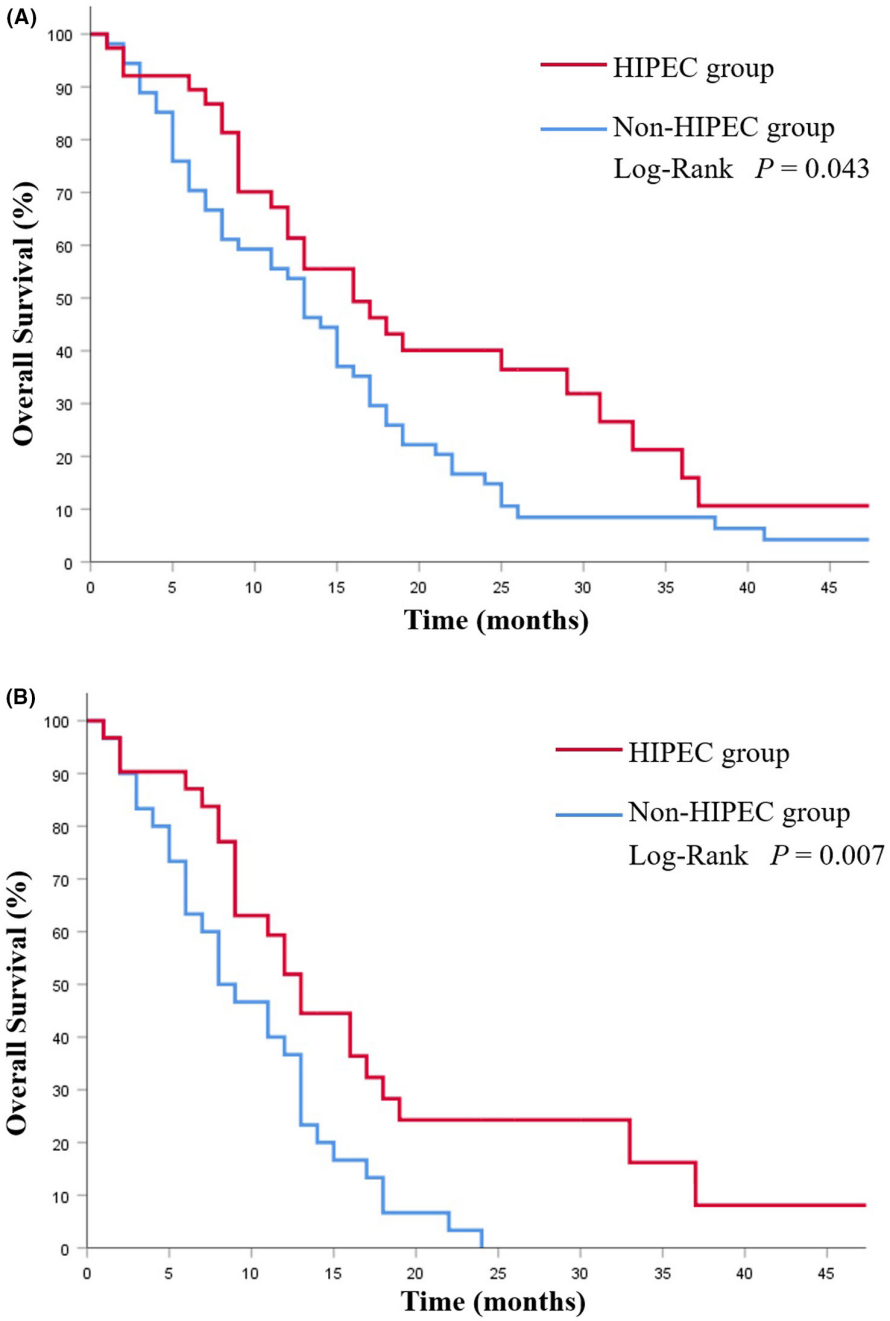
Kaplan–Meier analysis of overall survival in patients with or without HIPEC treatment. (A) OS in all patients; (B) OS in patients with peritoneal metastasis.

Thirty‐eight patients (NCS group 14 cases, CS group 24 cases; *p* = 0.056) received HIPEC treatment. The HIPEC group had a notable survival improvement (16.0 months vs.13.0 months; *p* = 0.043; Figure 5A). This difference was more pronounced in patients with peritoneal metastasis (13.0 months vs. 8.0 months; *p* = 0.007; Figure 5B).

### 3.4 Adverse events and postoperative complications

In this study, 26 out of 92 patients (28.26%) developed Grade 3–4 adverse events (NCS group16 cases, CS group 10 cases; *p* > 0.05). Among these, leucopenia/neutropenia (11.96%) and thrombocytopenia (2.17%) were the most frequent hematological toxic effects, while elevated serum AST levels (8.70%) and nausea (6.52%) were the most common non‐hematological toxic effects.

Eight patients (17.39%) in the CS group developed postoperative complications (Clavien‐Dindo grade II–III), which included four cases of pneumonia, three cases of intestinal obstruction, and one case of anastomotic stricture. All complications were successfully alleviated by conservative treatment.

### 3.5 Prognostic factors

According to univariate analysis, conversion therapy, peritoneal carcinomatosis, HIPEC treatment, and preoperative serum levels of CA199 and CA125 are significantly associated with survival. Multivariate analysis showed that conversion therapy (HR = 0.455; 95% CI 0.275–0.752; *p* = 0.002) and peritoneal metastasis (HR = 2.148; 95% CI 1.184–3.896; *p* = 0.012) were identified as independent predictors of OS (Table 4).

**TABLE 4:** Univariate and multivariate analysis of prognostic factors for OS.

Variable	Univariate		Multivariate	
	HR (95% CI)	*p‐*value	HR (95% CI)	*p‐*value
Age (≥50)	1.474 (0.920–2.359)	0.107		
Tumor size (≥5 cm)	1.458 (0.856–2.486)	0.165		
Bilateral ovarian metastases	1.089 (0.648–1.829)	0.748		
Conversion therapy	0.354 (0.225–0.557)	**<0.001**	0.455 (0.275–0.752)	**0.002**
HIPEC	0.629 (0.396–1.001)	**0.046**	0.597 (0.352–1.015)	0.057
cT‐stage (>T2)	1.171 (0.569–2.301)	0.646		
cN‐stage (N2‐3)	1.172 (0.732–1.877)	0.509		
Differentiation (poorly/undifferentiated)	1.473 (0.459–4.721)	0.515		
Ascites	1.107 (0.694–1.766)	0.670		
Signet‐ring cells	1.570 (0.955–2.581)	0.076		
Peritoneal carcinomatosis	2.383 (1.455–3.905)	**0.001**	2.148 (1.184–3.896)	**0.012**
CEA (>5 U/mL)	1.207 (0.730–1.993)	0.463		
CA199 (>37 U/mL)	1.949 (1.244–3.054)	**0.004**	1.507 (0.949–2.393)	0.082
CA125 (>35 U/mL)	1.927 (1.180–3.146)	**0.009**	1.567 (0.918–2.675)	0.100

Abbreviations: CA125, carbohydrate antigen 125; CA199, carbohydrate antigen199; CEA, carcinoembryonic antigen.

Values in bold indicate statistical differences.

Subgroup analysis showed that in the CS group, univariate analysis presented a significant association between ovarian metastasectomy before systemic chemotherapy, R0 resection, peritoneal carcinomatosis and survival. After adjustment for covariates on multivariate analysis, ovarian metastasectomy before systemic chemotherapy (HR = 0.339; 95% CI 0.143–0.799; *p* = 0.013), R0 resection (HR = 0.387; 95% CI 0.164–0.913; *p* = 0.030) and peritoneal carcinomatosis (HR = 2.308; 95% CI 1.087–4.902; *p* = 0.029) were identified as independent predictors of OS in patients underwent conversion therapy (Table 5).

**TABLE 5:** Univariate and multivariate analysis of prognostic factors for OS in CS group.

Variable	Univariate		Multivariate	
	HR (95% CI)	*p‐*value	HR (95% CI)	*p‐*value
Age (≥50)	1.952 (0.960–3.861)	0.065		
Tumor size (≥5 cm)	1.333 (0.615–2.887)	0.466		
Bilateral ovarian metastases	1.472 (0.569–3.805)	0.746		
HIPEC	0.574 (0.291–1.133)	0.109		
Ovarian metastasectomy before systemic chemotherapy	0.320 (0.150–0.682)	**0.003**	0.339 (0.143–0.799)	**0.013**
R0 resection	0.220 (0.103–0.417)	**<0.001**	0.387 (0.164–0.913)	**0.030**
cT‐stage (>T2)	2.263 (0.530–9.669)	0.270		
cN‐stage (N2‐3)	1.007 (0.481–2.107)	0.985		
Differentiation (poorly/undifferentiated)	2.792 (0.370–21.081)	0.319		
Ascites	1.155 (0.571–2.335)	0.689		
Signet‐ring cells	1.320 (0.573–3.041)	0.514		
Peritoneal carcinomatosis	2.286 (1.158–4.512)	**0.017**	2.308 (1.087–4.902)	**0.029**
CEA (>5 U/mL)	1.071 (0.465–2.464)	0.872		
CA199 (>37 U/mL)	1.610 (0.803–3.231)	0.180		
CA125 (>35 U/mL)	1.195 (0.605–2.361)	0.607		

Abbreviations: CA125, carbohydrate antigen 125; CA199, carbohydrate antigen199; CEA, carcinoembryonic antigen.

Values in bold indicate statistical differences.

## 4 DISCUSSION

Ovarian metastasis from GC is characterized by aggressive biological behavior and poor outcome. Palliative chemotherapy was used for such patients, however, with a response rate of only 12%–26% and a mOS of 7–11 months, its results were unsatisfactory.^6^, ^7^, ^11^ Currently, there is still lack of suitable treatment modality for GC patients with ovarian metastasis.

Up to date, some retrospective researches have shown that ovarian metastasectomy can improve the prognosis of these patients.^11^, ^13^, ^21^, ^22^ Cho et al. reported that patients who underwent ovarian metastasectomy combined with chemotherapy could have a better prognosis.^11^ Our previous study analyzed 152 GC patients with ovarian metastasis between 2005 and 2015, and the findings also showed that metastasectomy combined with chemotherapy was more effective than chemotherapy alone.^23^ The above studies suggest that for GC patients with ovarian metastasis, ovarian metastasectomy combined with chemotherapy can prolong the survival time; however, the role of the primary GC resection after ovarian metastasectomy and different modalities of conversion therapy warrants further exploration.

In this study, 92 patients with synchronous ovarian metastasis from GC were matched using a 1:1 PSM analysis. The mOS in the CS group shows a significant difference with which in the NCS group (19.0 months vs. 8.0 months; *p* < 0.001). Additionally, a total of 26 patients (56.52%) in the CS group achieved R0 resection, and the prognosis of them was significantly improved compared with those without R0 resection (26.0 months vs. 15.0 months; *p* = 0.003). Our findings show that the prognosis of these patients can be significantly improved by the conversion therapy model of chemotherapy combined with gastrectomy and ovarian metastasectomy, particularly in R0 resection cases.

Moreover, we compared the differences between conversion therapy modalities. Patients who underwent ovarian metastasectomy before systemic chemotherapy (CSa group) had a better R0 resection rate and longer OS than those who underwent simultaneous resection of primary GC and ovarian metastases after chemotherapy (CSb group; R0 resection rate: 73.08% vs. 35.00%, *p* = 0.016; OS: 29.0 months vs. 17.0 months, *p* = 0.002). These patients are often present with ascites or peritoneal metastasis, which are associated with a large tumor burden and poor chemosensitivity.^11^, ^24^, ^25^ Therefore, the resection of ovarian metastases can reduce the tumor burden and improve the efficacy of chemotherapy in some patients and thus may bring survival benefits.^23^, ^26^ In this study, compared with patients in the CSb group, patients in the CSa group had a better pathological response (*χ* ^2^ = 5.839, *p* = 0.028). Thus, for GC patients with synchronous ovarian metastases, particularly cases with large metastases and massive ascites, the conversion therapy mode of ovarian metastasectomy‐chemotherapy‐gastric tumor resection may be a better choice; however, the results need to be confirmed by further clinical studies.

Analysis of prognostic factors shows that peritoneal carcinomatosis is one of the independent predictors of OS. Ovarian metastasis from GC is often present with varying degrees of peritoneal metastasis, which often leads to ascites and ileus, and is associated with a poor prognosis.^12^, ^13^, ^19^, ^27^ There is currently no clear consensus on the treatment modality for GC patients with ovarian metastasis, especially for patients with peritoneal metastases. For these patients, cytoreductive surgery plus chemotherapy may offer a survival benefit as reported.^28^, ^29^ In our research, there are 61 cases combined with peritoneal carcinomatosis in GC patients with ovarian metastases. Compared with patients who received chemotherapy alone, those who underwent ovarian metastasectomy combined with gastrectomy presented a better survival (16 months vs. 8 months, *p* < 0.001), and further analysis presented that ovarian metastasectomy before systemic chemotherapy can achieve a better prognosis in such patients (18.0 months vs. 12 months, *p* = 0.017). Moreover, previous studies have shown that HIPEC can exert a potent antitumor effect by increasing contact between tumor lesions and chemotherapeutic agents while enhancing cytotoxicity through its thermal effect.^30^, ^31^ For GC patients with peritoneal metastasis, several studies have confirmed that HIPEC can improve their survival,^32^, ^33^, ^34^ and HIPEC has been reported as widely performed in patients with ovarian cancer.^35^, ^36^ In our study, HIPEC group showed a noteworthy improvement in survival (16.0 months vs. 13.0 months; *p* = 0.043), and further analysis revealed that the difference in prognosis was more significant in patients with peritoneal metastasis (13.0 months vs. 8.0 months; *p* = 0.007). Therefore, HIPEC can prolong the survival time of GC patients with synchronous ovarian metastasis, especially for patients with peritoneal metastases. However, more large sample researches are still required to further verify these results.

Our study also had some limitations. Above all, it was a single‐center retrospective study; PSM can reduce the selection bias, but it cannot be completely eliminated. Differences in some factors between the two groups, such as peritoneal metastasis, preoperative serum levels of CA199 and CA125 may affect the final results. Second, the overall sample size was small, and the conclusions need to be verified by more prospective clinical researches. However, as far as we know, this is the first PSM study on conversion therapy for GC patients with synchronous ovarian metastasis, which has important implications for future exploration of effective treatment models for such patients.

In conclusion, the conversion therapy mode of chemotherapy combined with surgical resection for patients with synchronous ovarian metastasis from GC is safe and effective, and can improve the prognosis of these patients. Moreover, resection of ovarian metastases before systemic chemotherapy and combined treatment with HIPEC could improve the therapeutic effect in such patients. In the future, more randomized controlled researches are needed to provide a basis for optimizing the treatment mode for GC patients with synchronous ovarian metastasis.

## AUTHOR CONTRIBUTIONS

**Jingquan Fang:** Conceptualization (equal); data curation (lead); formal analysis (lead); investigation (equal); methodology (equal); project administration (lead); resources (equal); software (lead); supervision (equal); writing – original draft (lead); writing – review and editing (lead). **Xingmao Huang:** Data curation (equal); software (equal); writing – review and editing (equal). **Xiangliu Chen:** Data curation (supporting); software (supporting). **Qi Xu:** Data curation (supporting). **Tengjiao Chai:** Data curation (supporting). **Ling Huang:** Data curation (supporting). **Han Chen:** Data curation (supporting); software (supporting). **Hang Chen:** Data curation (supporting); software (supporting). **Zeyao Ye:** Data curation (supporting). **Yian Du:** Data curation (supporting). **Pengfei Yu:** Conceptualization (lead); data curation (equal); formal analysis (equal); funding acquisition (lead); investigation (equal); methodology (lead); project administration (equal); resources (lead); supervision (lead); writing – review and editing (equal).

## ETHICS STATEMENT

The study was approved by the Institutional Ethics Committee of Zhejiang Cancer Hospital (no. IRB‐2022‐279) and informed consent has been waived by the Ethic Committee.

## Data Availability

The data that support the findings of this study are available on request from the corresponding author. The data are not publicly available due to privacy or ethical restrictions.
